# Ion channel molecular complexes in vascular smooth muscle

**DOI:** 10.3389/fphys.2022.999369

**Published:** 2022-08-26

**Authors:** Eric A. Pereira da Silva, Miguel Martín-Aragón Baudel, Manuel F. Navedo, Madeline Nieves-Cintrón

**Affiliations:** Department of Pharmacology, University of California, Davis, Davis, CA, United States

**Keywords:** l-type calcium channel, TRP channels, potassium channels, blood pressure, resistance arteries

## Abstract

Ion channels that influence membrane potential and intracellular calcium concentration control vascular smooth muscle excitability. Voltage-gated calcium channels (VGCC), transient receptor potential (TRP) channels, voltage (K_V_), and Ca^2+^-activated K^+^ (BK) channels are key regulators of vascular smooth muscle excitability and contractility. These channels are regulated by various signaling cues, including protein kinases and phosphatases. The effects of these ubiquitous signaling molecules often depend on the formation of macromolecular complexes that provide a platform for targeting and compartmentalizing signaling events to specific substrates. This manuscript summarizes our current understanding of specific molecular complexes involving VGCC, TRP, and K_V_ and BK channels and their contribution to regulating vascular physiology.

## Introduction

The diameter of small resistance arteries is a critical determinant of blood flow and tissue perfusion. The contractility of vascular smooth muscle (VSM) regulates arterial diameter. Multiple ion channels regulate VSM contraction by controlling membrane potential and the magnitude of intracellular calcium concentration [Ca^2+^]_i_ (Knot and Nelson, 1995). In VSM, voltage-dependent L-type Ca_V_1.2 (LTCCs) channels are the main Ca^2+^ influx pathway for contraction (Ghosh et al., 2017), and a role for T-type channels regulation of VSM excitability is also described (Cribbs, 2006; Abd El-Rahman et al., 2013). Transient receptor potential (TRP) channels contribute to vascular function by regulating membrane potential, contraction, and myogenic tone development (Brayden et al., 2008). Voltage (K_V_) and Ca^2+^-activated K^+^ (BK) channels provide a negative-feedback regulation of VGCC activity, hence Ca^2+^ influx and contraction, by modulating VSM membrane potential. Different stimuli within the body, including variations in pressure, vasoactive substances released from endothelial cells, and nerve terminals, modulate contraction by initiating cellular signaling that impinges on the function of these ion channels.

Signaling complexes permit efficient transduction of the many signals received with the specificity necessary to support function. VGCC, K^+^, and TRP channels form complexes with key proteins (e.g., ion channels, GPCR, signaling molecules) that modulate their function and, in doing so, regulate VSM contractility. Scaffold proteins, such as AKAP5, have been shown to facilitate molecular complex formation with signaling and effectors proteins in VSM. For more extensive reviews on the different ion channels, readers are directed to (Earley and Brayden, 2015; Ghosh et al., 2017; Tykocki et al., 2017). Here we will summarize current knowledge on specific molecular complexes involving LTTC, TTCC, TRP, K_V,_ and BK channels and their contribution to regulating vascular physiology.

### Voltage-gated calcium channels

Voltage-gated Ca^2+^ channels (VGCC) are found in various cell types throughout the body. Changes in membrane potential activate these channels, leading to Ca^2+^ influx and the regulation of many physiological processes (Catterall, 2011). VGCC comprises a family of ten members, subdivided into three major subfamilies, Ca_V_1, Ca_V_2, and Ca_V_3, based on their biophysical properties (Catterall, 2000; Catterall, 2011). Members of the Ca_V_1 and Ca_V_2 families have been identified in VSM (Tykocki et al., 2017). The Ca_V_1 family activates at depolarized membrane potential and is characterized by large conductance and long openings, hence their name L-type Ca^2+^ channels (LTCC). Meanwhile, the Ca_V_2 or T-type (TTCC) family of VGCC has tiny currents that activate at more negative potentials than the LTCC and inactivate fast (Catterall, 2011). Of relevance to this review is the LTCC, which is necessary for the VSM myogenic contraction (Moosmang et al., 2003), and the TTCC that emerging data suggest participate in arterial tone regulation (Cribbs, 2006; Abd El-Rahman et al., 2013; Harraz et al., 2015a). In the following section, we summarize the contributions of these channels to VSM physiology, emphasizing their participation in the signaling domains that facilitate the regulation of VSM excitability.

### Ca_V_1.2 signaling complexes in VSM

L-type calcium channel Ca_V_1.2 is the main entryway for Ca^2+^ in vascular smooth muscle contributing Ca^2+^ for contraction and gene expression. Indeed, Ca^2+^ influx through Ca_V_1.2 is necessary for pressure-induced contraction (i.e., myogenic tone), and studies suggest that about 50% of phenylephrine-induced contraction is due to Ca^2+^ influx via these channels (Moosmang et al., 2003). Moreover, Ca^2+^ influx via Ca_V_1.2 channels is linked to transcriptional regulation in VSM (Wamhoff et al., 2004; Wamhoff et al., 2006; Kudryavtseva et al., 2013). The altered function of the Ca_V_1.2 channels is associated with contractility and gene expression changes. For instance, studies associate changes in Ca_V_1.2 function with increased myogenic tone in conditions such as hypertension (Nieves-Cintron et al., 2008; Navedo et al., 2010a) and diabetes (Navedo et al., 2010b; Nystoriak et al., 2014; Nystoriak et al., 2017a).

LTCCs are heteromeric complexes of α1-, β, and α2δ subunits (Catterall, 2000). The *α*1-subunit, which forms the ion conduction pore, consists of four homologous domains (I-IV), each domain having six transmembrane segments (S1–S6) linked by three intracellular loops, and intracellular amino and carboxy-terminal. The homologous transmembrane segment S6 and the loop between S5 and S6 of each homologous domain form the ion conduction pore. Voltage sensitivity is provided by transmembrane segment S1-S4 (Catterall, 2011). β and α2δ subunits are auxiliary subunits that have been shown to contribute to membrane trafficking and regulate the channel biophysics (De Jongh et al., 1990; Singer et al., 1991; Gao et al., 1999; Dolphin, 2003; Catterall, 2011). Among the four known subtypes (β1 - β4), β3 is the principal subunit in VSM (Murakami et al., 2000; Kharade et al., 2013). The α2δ subunit arises from a single gene; subsequent posttranslational processing produces an extracellular α2 and membranal δ subunits that associate via a disulfide bridge to form a functional subunit (Gurnett et al., 1996; Arikkath and Campbell, 2003). Three different α2δ delta isoforms have been identified (α2δ1 - α2δ3) (Klugbauer et al., 1999). The α2δ1subunit was critical for membrane expression of the Ca_V_1.2 α1c in VSM from rat cerebral artery (Bannister et al., 2009).

Alterations in the subunit expression profiles have been linked to changes in physiology. For example, studies report increased expression of Ca_V_1.2 α1c subunit in VSM in models of high blood pressure, including genetic models of hypertension (Pratt et al., 2002; Pesic et al., 2004). Moreover, increased expression of β3 subunit contributes to the upregulation of Ca_V_1.2 α1c subunit membrane expression in VSM from animal models of hypertension owing to its role in the channel trafficking (Kharade et al., 2013). Elevation of the α2δ1 subunit expression during hypertension was also reported and linked with higher Ca_V_1.2 membrane surface expression and currents (Bannister et al., 2012). Changes in the expression of LTCC subunits have also been reported in a genetic model of hypertension (BPH mice) (Tajada et al., 2013). However, while several studies report increases in the expression of the Ca_V_1.2 α1c subunit in hypertension (Pratt et al., 2002; Pesic et al., 2004; Sonkusare et al., 2006), the study by Tajada et al. shows a decrease in Ca_V_1.2 α1c expression in mesenteric artery VSM form BPH mice relative to normotensive control mice (Tajada et al., 2013). Accordingly, mesenteric VSM from BPH mice showed a reduction in Ca_V_1.2 α1c and a change in the expression profile of the accessory subunits. The authors proposed that in normotensive BPN mice, Ca_V_1.2 currents were carried by α1c/β3/α2δ, whereas in BPH mice, the current was likely mediated by channels composed of α1c/β2/α2δ subunits. This discrepancy could be due to the hypertension model used (e.g., angiotensin-induced vs. genetic hypertension), the species (rat vs. mice), and vascular beds. Nevertheless, the studies highlight the relevance of the accessory subunits in regulating LTCC activity and VSM contractility.

**FIGURE 1:**
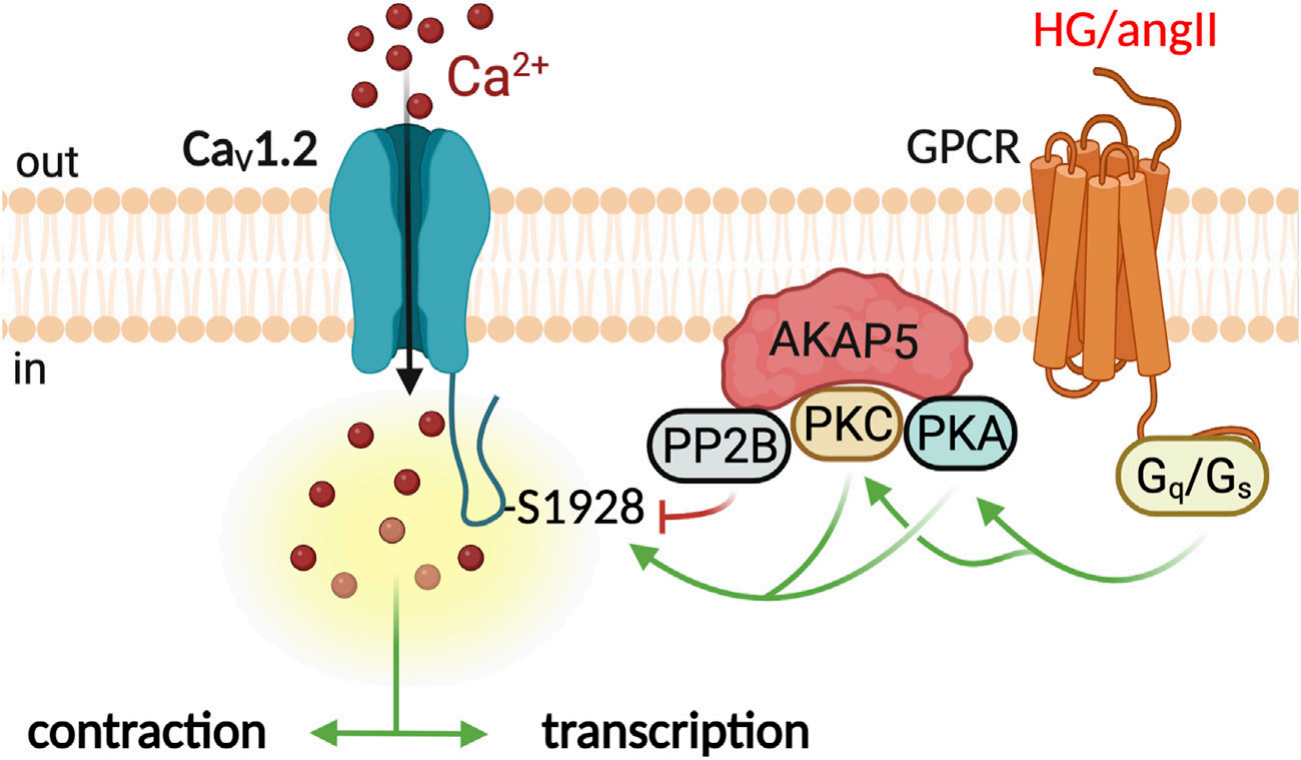
Ca_V_1.2 molecular complex in the regulation of VSM contraction and transcription. Ca^2+^ influx through Ca_V_1.2 channels is essential for the pressure-induced contraction of the VSM (Moosmang et al., 2003). Activation of G_s_/PKA signaling by high glucose or G_q_/PKC by angiotensin II potentiate Ca_V_1.2 activity (Navedo et al., 2008; Navedo et al., 2010b; Prada et al., 2019). The scaffolding protein AKAP5 facilitates PKA/PKC phosphorylation of Ca_V_1.2 channels at S1928 (Nystoriak et al., 2017a). The phosphatase PP2B opposes phosphorylation dampening Ca_V_1.2 potentiation. Graphics created with BioRender.com.

In VSM, the Ca_V_1.2 channel forms molecular complexes with signaling components including receptors, enzymes, and effector proteins orchestrated by scaffolds proteins, such as the A-kinase anchoring protein 5 (AKAP5) (Tykocki et al., 2017). AKAPs are structurally diverse intracellular scaffolding proteins that bind PKA, PKC, calcineurin (PP2B), and Ca_V_1.2, thus facilitating regulation of the channel by these proteins (Colledge and Scott, 1999; Langeberg and Scott, 2005). Optical recording of Ca^2+^ influx revealed that protein kinases and phosphatases modulate CaV1.2 activity in an AKAP-dependent manner in VSM (M Nieves-Cintron et al., 2018) (Figure 1). Fluorescent signals elicited by LTCC openings (Ca_V_1.2-sparklets) were visualized using TIRF microscopy. Contrary to expectation, not all Ca_V_1.2 channels had a similar open probability. Subpopulations of channels showed stochastic transient openings (low activity sparklets). In contrast, other channels displayed events with prolonged available time produced by activating two or more channels that generated areas of almost continuous calcium influx (Navedo et al., 2005). This high-activity mode, termed persistent sparklets, required PKC activity and AKAP5 expression (Navedo et al., 2006). Moreover, the phosphatase PP2B is part of the molecular complex, and its activity limits Ca_V_1.2-persistent sparklets (Navedo et al., 2006). Persistent Ca_V_1.2-sparklets contributed to approximately 50% of Ca^2+^ influx through LTCC (Amberg et al., 2007), highlighting the relevance of these events and the macromolecular complex that regulates them to VSM physiology.

Ca_V_1.2-sparklets events are of higher frequency and amplitude in VSM from hypertensive mice than in corresponding control mice. In the angiotensin II-induced hypertension model, higher Ca_V_1.2-sparklets required activation of PKC signaling (Navedo et al., 2008). Higher Ca_V_1.2 sparklets increased arterial wall Ca^2+^, tone, and blood pressure. Interestingly, angiotensin II failed to elicit an increase in Ca_V_1.2-sparklets in AKAP5 knockout mice (Navedo et al., 2008). Moreover, these mice did not develop hypertension in response to angiotensin infusion (Navedo et al., 2008). Thus, angiotensin II increases arterial tone and blood pressure by stimulating Ca_V_1.2 sparklets locally in an AKAP5-dependent manner. VSM from murine models of diabetes also shows elevated Ca^2+^ sparklet activity, and AKAP5 is also central to the signaling events leading to higher Ca_V_1.2 sparklet activity during diabetic hyperglycemia (Navedo et al., 2010b; Nieves-Cintron et al., 2021). AKAP5 orchestrates a signaling module that includes the purinergic receptor P2Y_11_, adenylyl cyclase 5, PKA, and the Ca_V_1.2 subunit (Prada et al., 2019; Syed et al., 2019; Prada et al., 2020) (Figure 1). Activating this signaling module increases Ca_V_1.2 activation and arterial contractility during diabetes. The fact that PKA was required for Ca_V_1.2-sparklets during diabetes is surprising as this kinase is associated with vasodilation. It also highlights the exquisite level of signal specificity achieved by segregating receptors, signaling enzymes, and effector proteins (Colledge and Scott, 1999; Langeberg and Scott, 2005) into macromolecular complexes.

### T-type calcium channel signaling complex in VSM

TTCC α1 subunit topology is similar to that of other VGCC. However, no auxiliary subunits have been co-purified with the TTCC, but studies suggest that TTCC activity may be modified by other proteins, including the LTCC auxiliary subunits (Perez-Reyes, 2006; Dolphin, 2016). TTCC subunits Ca_V_3.1, Ca_V_3.2, and Ca_V_3.3 have been identified in VSM from several vascular beds, and emergent evidence points toward a contribution of these channels to the regulation of the VSM reactivity (Cribbs, 2006; Abd El-Rahman et al., 2013). TTCCs have been shown to contribute to vasoconstrictor responses in mesenteric arterioles (Gustafsson et al., 2001), skeletal muscle arteries (VanBavel et al., 2002), and retinal arteriolar (Fernandez et al., 2015) VSM. In cerebral VSM from rats and mice, Ca_V_3.1 and Ca_V_3.2 carry the nifedipine insensitive component of Ba^2+^ currents (Nikitina et al., 2007; Abd El-Rahman et al., 2013; Harraz et al., 2015b). Human cerebral arteries express Ca_V_3.2 however the Ca_V_3.1 described in rodents is replaced by the Ca_V_3.3 isoform in these arteries (Harraz et al., 2015a). Ca_V_3.1 and Ca_V_3.3 activity were found to contribute to the pressure-induced constriction (Harraz et al., 2014; Harraz et al., 2015b). Interestingly, selective inhibition of Ca_V_3.2 channels with micromolar Ni^2+^ concentration elicited depolarization and constriction of wild-type rat cerebral arteries (Harraz et al., 2014). Electron tomography of rat cerebral VSM showed microdomains consisting of caveolae and sarcoplasmic reticulum (SR). Immunogold labeling localized Ca_V_3.2 and RyR to these microdomains (Harraz et al., 2014). The studies proposed that caveolae facilitate a Ca^2+^ signaling network that enables the coupling of Ca_V_3.2 mediated Ca^2+^ influx with activation of RyR receptors leading to activation of Ca^2+^-activated potassium channels contributing to membrane potential hyperpolarization (Harraz et al., 2014; Harraz et al., 2015a; Harraz et al., 2015b). Thus, the segregation into a microdomain allows Ca_V_3.2 to modulate arterial tone by regulating the RyR-BK channels axis (Harraz et al., 2014; Harraz et al., 2015a).

### TRP channels signaling complexes in VSM

TRP channels are a superfamily of cation channels encoded by 28 genes. The superfamily is divided into six subfamilies based on sequence homology, which include TRPC (canonical), TRPV (vanilloid), TRPM (melastatin), TRPP (polycystin), TRPA (ankyrin), and TRPML (mucolipin) channels (Earley and Brayden, 2015). TRP channels comprise six membrane-spanning segments (S1-S6) with intracellular amino and carboxy terminals. Electron cryomicroscopy studies of the TRPV1 channel suggest a symmetrical four-fold arrangement with the S5 and S6 loops forming the ion path (Liao et al., 2013). VSM cells express several TRP channels, which contribute to regulating membrane potential, contraction, and myogenic tone development. Some TRP channels translate mechanosensitive G-coupled signaling into changes in membrane potential in VSM cells (Earley and Brayden, 2015), thus contributing to artery autoregulation. Recent studies uncovered a role for specific TRP channels in signaling networks and molecular complexes that contribute to regulating contractile state. Here we summarize these studies, emphasizing well-characterized TRP channel signaling complexes contributing to the regulation of VSM excitability.

### TRPC3 & TRPC6

**FIGURE 2:**
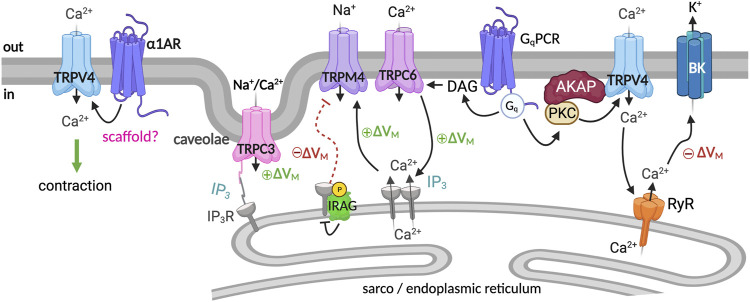
TRP channel signaling complex regulates VSM contractility. Several TRP channels are involved in regulating VSM excitability. Caveolae scaffolding protein caveolin-1 (cav-1) co-localizes the IP_3_R1 and TRPC3 channels close to each other. IP_3_—induced coupling of IP_3_R1 and TRPC3 leads to TRPC3 activation and VSM depolarization (Xi et al., 2008; Adebiyi et al., 2011). TRPC6/TRPM4, PLCγ1, and IP_3_R are part of a stretch-sensing signaling molecular complex that translates pressure-induced mechanical stimuli into VSM membrane depolarization (Reading and Brayden, 2007; Gonzales et al., 2014). PLC activation generates IP_3_, which sensitizes IP_3_Rs to TRPC6-mediated Ca^2+^ influx boosting SR Ca^2+^ release through IP_3_R channels to activate the TRPM4 channel causing cell depolarization and contraction (Gonzales et al., 2014). The NO/cGMP/PKG axis signals through IRAG to inhibit IP_3_R-mediated Ca^2+^ release from the SR, blunting the TRPM4 activity (Ali et al., 2021). TRPV4 is suggested to contribute to contraction and relaxation via the formation of distinct molecular complexes that are spatially segregated (Chen et al., 2022). The TRPV4/α1AR axis leads to agonist-induced contraction (Chen et al., 2022), whereas the TRPV4/BK complex hyperpolarizes the VSM membrane potential (Earley et al., 2005; Chen et al., 2022). Graphics created with BioRender.com.

In VSM cells, the plasmalemmal canonical transient receptor potential 3 (TRPC3) channels decode G-protein coupled receptor (GPCR) signaling into a cation current that elicits cell depolarization (Earley and Brayden, 2015). Indeed, in VSM, TRPC3 channels are implicated in cerebral artery constriction in response to GPCR agonists such as angiotensin II and endothelin-1 (ET-1) (Reading et al., 2005; Xi et al., 2008; Earley and Brayden, 2015). Intriguingly, TRPC3 channels in VSM are activated by GPCR-phospholipase C (PLC) signaling independently of SR Ca^2+^ release and PKC (Xi et al., 2008). The agonist IP_3_ was shown to constrict rat (Sprague-Dawley (SD), male and female) cerebral arteries by facilitating coupling between type 1 inositol 1,4,5-trisphosphate receptor type 1 (IP_3_R1) and TRPC3 channels (Xi et al., 2008). Further studies showed that in cerebral artery (SD, male) VSM, the caveolae scaffolding protein caveolin-1 (cav-1) co-localizes the IP_3_R1 and TRPC3 channels close to each other (Adebiyi et al., 2011). This signaling complex allows IP_3_—induced coupling of IP_3_R1 and TRPC3, leading to TRPC3 activation and generation of cation current that depolarizes VSM, thus eliciting contraction (Adebiyi et al., 2011) (Figure 2).

TRPC6 channels also contribute to regulating pressure-induced VSM contraction (Welsh et al., 2002). Like TRPC3, TRPC6 channels are activated by GPCR-PLC agonists such as angiotensin II via mechanisms requiring diacylglycerol but independent of PKC (Helliwell and Large, 1997; Saleh et al., 2006). Studies using cerebral arteries from male SD rats suggest that TRPC6 channels are part of a stretch-sensing signaling network, including PLCγ1, TRPM4, and IP3Rs that translate pressure-induced mechanical stimuli into membrane depolarization of cerebral VSM (Gonzales et al., 2014) (Figure 2). According to the proposed model, initiation of PLCγ1 signaling generates IP_3_, which sensitizes IP_3_Rs to TRPC6-mediated Ca^2+^ influx. This process boosts SR Ca^2+^ release through IP_3_R, activating TRPM4 channels, cell depolarization, and artery constriction (Gonzales et al., 2014).

### TRPM4

Increases in intraluminal pressure depolarize VSM and constrict cerebral arteries partly via the activation of the TRPM4 channel (Brayden et al., 2008). TRPM4 channels are selective for monovalent cations. Accordingly, TRPM4 channels are activated by Ca^2+^ but do not permeate this ion. Calmodulin binding sites have been identified on the C-terminus of the TRPM4 channel, which influences the sensitivity to Ca^2+^ (Nilius et al., 2005). Moreover, TRPM4 Ca^2+^ sensitivity is regulated by ATP and PKC-mediated phosphorylation of putative serine residues in the carboxy-terminal (Nilius et al., 2005). Suppressing TRPM4 expression in rat pial arteries compromises the ability of the arteries to regulate blood flow in response to changes in the mean arterial pressure (MAP) (Reading and Brayden, 2007), highlighting the physiological relevance of the channel for blood flow autoregulation. In rat’s cerebral VSM, TRPM4 channels are activated by local IP_3_R-mediated increase in [Ca^2+^]i (Gonzales et al., 2010). As mentioned above, with TRPC6, TRPM4 channels are part of a mechanosensitive signaling complex whereby IP3 is generated by activating GPCR-PLCγ1 signaling, which sensitizes IP_3_Rs to TRPC6-Ca^2+^ influx boosting IP3R-SR Ca^2+^ release to activate TRPM4 (Gonzales et al., 2014) (Figure 2). By regulating TRPM4 activity, this signaling complex may regulate pressure-induced constriction in cerebral arteries.

Intriguingly, a recent study suggests that the tissue soluble gaseous vasodilator nitric oxide could relax arteries by inhibiting the activity of TRPM4 channels (Ali et al., 2021) (Figure 2). NO is produced by endothelial cells and exerts its vasodilatory action by increasing soluble cyclic guanosine monophosphate (cGMP) and stimulating the activity of cGMP-activated protein kinase (PKG) (Lincoln et al., 1985; Carvajal et al., 2000). Using male and female C57BL/6J cerebral arteries, a mechanism was described involving the formation of a signaling complex on the SR of VSM comprised of PKG, IP_3_Rs, and IP_3_R-associated cGMP-kinase substrate (IRAG) (Ali et al., 2021). IRAG is a regulator of IP_3_-triggered Ca^2+^ release found to be complex with IP_3_Rs in the VSM (Geiselhoringer et al., 2004). Phosphorylation of IRAG by activation of the NO/cGMP/PKG axis was found to inhibit IP_3_R-mediated Ca^2+^ release from the SR (Geiselhoringer et al., 2004; Schlossmann and Desch, 2011). As a result of IP_3_R inhibition and concomitant SR Ca^2+^ release, TRPM4 channel activity is blunted. This leads to a reduction in TRPM4-mediated membrane depolarization and voltage-dependent Ca^2+^ channel activity leading to vasodilation (Ali et al., 2021). The formation of this NO/cGMP/PKG/IRAG signaling complex allows for distinct regulation of TRPM4 channel activity and, therefore, VSM membrane potential and contractile state.

### TRPV4

VSM cells express transient receptor potential vanilloid 4 (TRPV4) channels. TRPV4 are Ca^2+^ permeable, nonselective cation channels, and their activation results in Ca^2+^ influx (Brayden et al., 2008). Interestingly, although the magnitude of the calcium influx through TRPV4 channels is significant, TRPV4 activity is linked to VSM relaxation (Earley et al., 2005). This has been attributed to relatively low basal TRPV4 activity and the formation of a signaling complex with ryanodine receptors (RyRs) and large-conductance Ca^2+^-activated potassium (BK) channels (Earley et al., 2005) (Figure 2). In cerebral and cerebellar arteries from male SD rats, activation of TRPV4 by endothelium-derived signals elicits Ca^2+^ influx that activates RyRs (Earley et al., 2005). RyR activation results in SR Ca^2+^ release (i.e., Ca^2+^-spark), coupled with the opening of BK channels at the plasma membrane of VSM cells. Activating the BK channels leads to VSM membrane hyperpolarization, decreased voltage-dependent Ca^2+^ channel activity, and intracellular Ca^2+^ concentration [Ca^2+^]_i,_ thereby causing VSM relaxation (Knot et al., 1998).

Studies using cerebral arteries from rats, C57BL/6J, and AKAP150^−/−^ mice suggest that in VSM, AKAP5 targeted PKCα facilitates regulation of TRPV4 channels by GqPCR signaling (Mercado et al., 2014; Tajada et al., 2017). Interestingly, a new study using mice mesenteric and human paraspinal muscle arteries submits that TRPV4 channels form spatially separated complexes with α1 adrenergic receptor (α1AR) and the BK channel, allowing them to respond differentially to different physiological stimuli (Chen et al., 2022). In this model, TRPV4 channels in proximity to the α1AR are activated by α1AR/GqPCR signaling and contribute to agonist-induced vasoconstriction. In contrast, pressure-induced activation of the TRPV4/BK channel axis opposes contraction (Chen et al., 2022) (Figure 2). The TRPV4/α1AR signaling axis was elevated in hypertension, whereas the TRPV4/BK complex was decreased, highlighting a potential new therapeutic target for hypertension. It will be important to determine if different scaffolds are responsible for the distinct spatial segregation of signaling components. The referenced studies suggest that forming distinct signaling complexes allows a diversity of the regulation and function of plasmalemmal TRP activity with profound consequences for VSM membrane potential and contractile state (Figure 2).

### K^+^ channels

K_V_ channels are a varied group of membrane proteins comprising at least 12 families, namely K_V_1-K_V_12 (Gutman et al., 2005). Structurally, K_V_ channels are formed by tetrameric assembly of alpha subunits, which form the ion conduction pore and auxiliary beta subunits (Choe et al., 1999). The tetramer’s subunit composition and inclusion of auxiliary beta subunits provide functional and pharmacological diversity (Zhong et al., 2010a; Kilfoil et al., 2013; Tykocki et al., 2017). VSM expresses multiple K_V_ channel subunits, including members of the K_V_1 (K_V_1.1, K_V_1.2, K_V_1.3, K_V_1.5, K_V_1.6) (Cheong et al., 2001; Albarwani et al., 2003; Amberg et al., 2003; Tykocki et al., 2017), and K_V_2 (K_V_2.1) (Amberg and Santana, 2006; Tykocki et al., 2017) families. Members of the K_V_7 (K_V_7.1–5) (Zhong et al., 2010b) and K_V_9 (K_V_9.3) (Zhong et al., 2010a; Tykocki et al., 2017) have also been identified. Homomeric and heteromeric channels consisting of an assembly of distinct alpha subunits with auxiliary K_V_β have been found. For example, heteromultimeric channels consisting of K_V_1.2-K_V_1.5 subunits have been shown to contribute to vascular K_V_ currents in VSM (Kerr et al., 2001; Albarwani et al., 2003; Plane et al., 2005). Heteromers of K_V_2.1 and silent K_V_9.3 subunits have been identified in VSM from rat cerebral arteries that contribute to regulating arterial diameter (Zhong et al., 2010a). Expression of K_V_6.3 silent subunit was reported in mesenteric VSM from hypertensive mice (Moreno-Dominguez et al., 2009). Studies in murine coronary microvasculature show an association between K_V_1.5 and auxiliary K_V_β1 and K_V_β2 (Nystoriak et al., 2017b). The study suggests that K_V_β2 facilitates K_V_1.5 membrane trafficking in coronary VSM (Nystoriak et al., 2017b). K_V_ channels control VSM contraction by regulating membrane potential and the magnitude of the VGCC-mediated Ca^2+^ influx (Knot and Nelson, 1995; Nystoriak and Navedo, 2017). Thus, mechanisms that regulate K_V_ activity will impact VSM membrane potential, contractility, and ultimately arterial tone and diameter.

**FIGURE 3:**
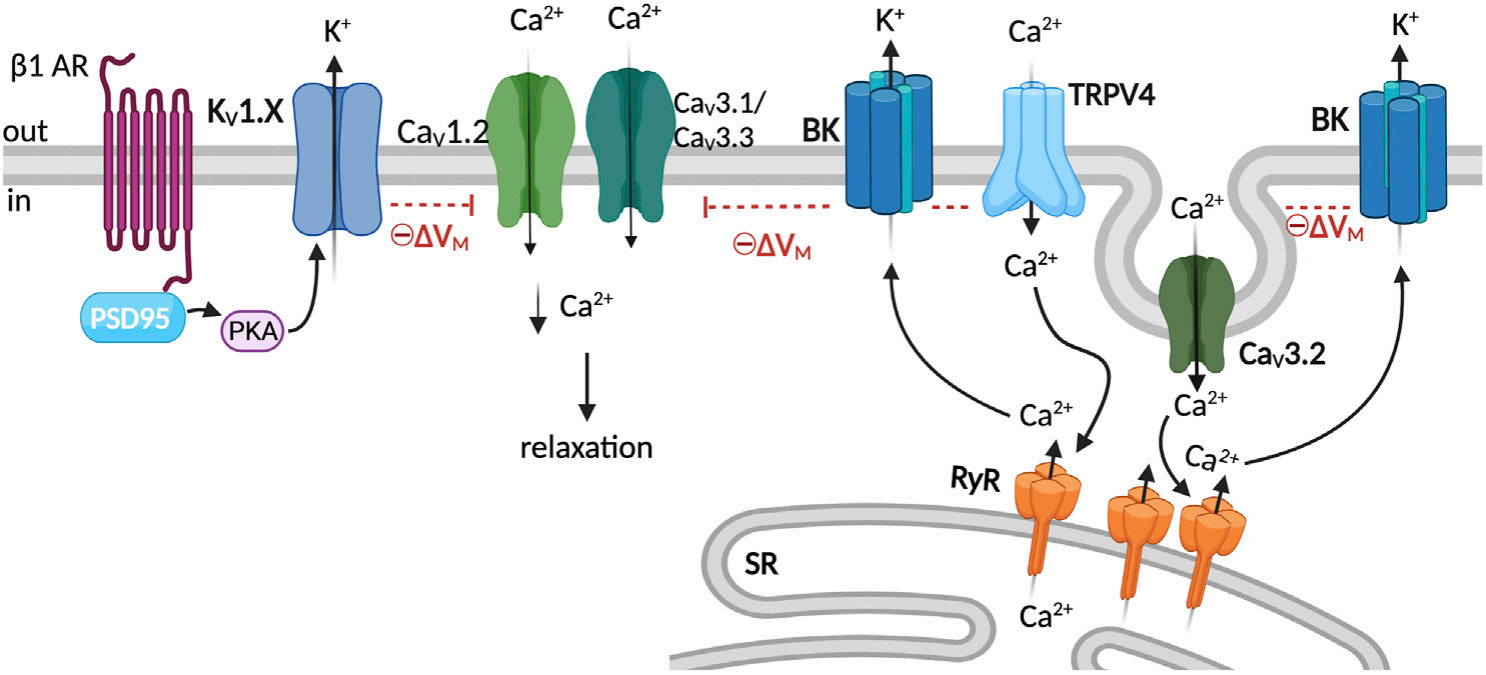
K_V_1 and BK complexes and membrane potential regulation. K^+^ channel activity provides negative-feedback regulation of membrane potential and limits Ca_V_1.2 activity leading to VSM relaxation (Nelson, 1992). The scaffolding protein PSD95 facilitates the regulation of K_V_1 channels by β1AR/PKA signaling (Moore et al., 2015). BK channels are activated by the release of Ca^2+^ from the SR through RyR (Ca^2+^ sparks) (Jaggar et al., 1998). Ca^2+^ influx through TRPV4 channels activates the RyR/BK channel axis, which hyperpolarizes VSM membrane potential (Earley et al., 2005). Caveolae facilitate spatial proximity between Ca_V_3.2 and RyR, Ca_V_3.2-mediated Ca^2+^ influx prompts RyR, and the resultant Ca^2+^ sparks activate BK channels (Harraz et al., 2014; Hashad et al., 2018), which hyperpolarizes VSM membrane potential and limits VGCC activity leading to relaxation. Graphics created with BioRender.com.

VSM K_V_ channel activity is modulated by intracellular signaling (Ko et al., 2010). For example, several vasoconstrictors are shown to partially constrict arteries via PKC-dependent inhibition of K_V_ channel activity (Aiello et al., 1996; Clement-Chomienne et al., 1996; Hayabuchi et al., 2001). In addition to PKC, the Src tyrosine kinase pathway has been shown to blunt K_V_ activity and contract rat mesenteric arteries (Sung et al., 2013). On the other hand, protein kinase A (PKA) is associated with K_V_ potentiation and vasodilation (Tykocki et al., 2017). For instance, β-adrenergic receptor agonists that engage adenyl cyclase (AC)/cAMP/PKA signaling promote 4-AP-sensitive K_V_ channel activity in VSM and vasodilation (Aiello et al., 1995; Cole et al., 1996; Satake et al., 1996; Berwick et al., 2010). Despite the evidence of intracellular signaling regulation of K_V_ activity and VSM contractility, the formation of macromolecular complexes that could facilitate this regulation is relatively unknown. Recent evidence, however, supports the role of the scaffolding protein postsynaptic density 95 (PSD95) in facilitating the regulation of K_V_ channels by β-adrenergic signaling (Joseph et al., 2011; Moore et al., 2014; Moore et al., 2015). As its name implies, PSD95 is found at the postsynaptic density, where it facilitates the formation of macromolecular complexes between receptors, ion channels, and signaling molecules (Won et al., 2017). PSD95 was found to colocalize with the β1-adrenergic receptor in VSM and to enable phosphorylation of K_V_1 channels by PKA in rat cerebral VSM (Joseph et al., 2011; Moore et al., 2014). Thus, in cerebral VSM, PSD95 facilitates β-adrenergic-mediated vasodilatory regulation via the formation of a macromolecular complex with K_V_1 channels (Moore et al., 2015; Rhee and Rusch, 2018) (Figure 3). Whether other scaffolds are involved in K_V_ channel regulation in VSM is an area for further research development in VSM.

### BK-channels

Large-conductance Ca^2+^-activated potassium channels (BK) are critical regulators of VSM contractility by tonically regulating membrane potential (Jaggar et al., 1998). BK channels are activated by calcium and membrane depolarization (Latorre et al., 2017). The channel comprises tetrameric assemblies of pore-forming alpha subunits, which harbor the voltage sensor and cytosolic calcium-binding sites (Ko et al., 2008; Latorre et al., 2017). The BKα subunit assembles with auxiliary beta and gamma subunits. Four β (β1-β4) and four γ isoforms (γ1-γ4) have been identified so far (Li and Yan, 2016). The β1 subunit is the main β subunit isoform expressed in VSM cells; it increases BK calcium sensitivity and alters the biophysical properties of the channel (Brenner et al., 2000). The γ1 subunit has been reported in rat cerebral VSM (male SD) cells and suggested to increase the BK channel voltage sensitivity (Evanson et al., 2014). BK channels are activated by the simultaneous release of SR Ca^2+^ via RyRs in close apposition to VSM plasmalemma BK channels (Nelson et al., 1995) (Figure 3). Functional coupling between BK channels and RyR has been extensively characterized in VSM as a critical mechanism regulating arterial diameter (Nelson et al., 1995; Perez et al., 1999; Fan et al., 2019). Moreover, studies in mice mesenteric and human cerebral VSM suggest that BK channels are part of a Ca^2+^ signaling microdomain that includes caveolae, T-type Ca_V_3.2, and RyRs (Harraz et al., 2014). Caveolae facilitates the coupling of Ca_V_3.2 channel activity with RyR activation (Hashad et al., 2018), and BK channels within the signaling domain are activated by the Ca^2+^ sparks (Figure 3). As described above, BK channels are also suggested to be in a signaling complex with TRPV4 and RyRs, which promote BK channel activation in response to changes in the intraluminal pressure (Earley et al., 2005).

## Conclusion

Small resistance arteries respond to different stimuli by adjusting their diameter to meet the tissue perfusion needs. The adjustments in the arterial diameter of small resistance arteries and arterioles are largely determined by the contractile state of VSM lining the walls of arteries (Knot and Nelson, 1995; Knot and Nelson, 1998; Cole et al., 2005). VSM contractility is dependent on the interplay of different ionic conductance that controls membrane potential and the level of intracellular [Ca^2+^]_i_. Different stimuli within the body engage signaling mechanisms that regulate these ion channels and, in turn, modulate vascular function. Here, we have provided an overview of our current knowledge of specific molecular complexes involving VGCC, TRP, and K_V_ and BK channels emphasizing well-characterized signaling complexes contributing to the regulation of VSM excitability. The formation of macromolecular complexes could provide a way for ion channels to respond differently to specific stimuli by bringing signaling generators and effectors within proximity to discrete regions of the cell. Determining whether similar complexes exist in different vascular beds and their contribution to VSM contractility in health and disease is an area of opportunity in the vascular field. Understanding the specific mechanism that governs the formation of these molecular complexes will provide novel strategies for developing therapeutics with enhanced specificity in the fight against cardiovascular disease.
